# Free-breathing 3D cardiac function with accelerated magnetization transfer prepared imaging

**DOI:** 10.1186/1532-429X-16-S1-P63

**Published:** 2014-01-16

**Authors:** Eric M Schrauben, Oliver Wieben, Kevin M Johnson

**Affiliations:** 1 Medical Physics, University of Wisconsin - Madison, Madison, Wisconsin, USA; 2 Radiology, University of Wisconsin - Madison, Madison, Wisconsin, USA

## Background

3D cardiac MRI has long held promise for improved heart coverage, higher resolution, and reduced sensitivity to poor breath-hold reproducibility. However, its use has been limited by reduced blood pool to myocardium contrast for spoiled and balanced steady-state free precession (bSSFP) implementations. T2-preparation techniques [1] are capable of increasing contrast but are unfortunately limited by lengthy preparation periods and resulting scan inefficiencies. In this work, we develop a paradigm for high contrast 3D cardiac function that relies on the alternative use of magnetization transfer (MT) preparation [2] combined with accelerated 3D spoiled gradient echo imaging (SPGR).

## Methods

An off-resonance RF pulse was interleaved with whole-heart, respiratory gated 3D radial SPGR sampling [3]. Simulations and phantom scans were performed to optimize MT saturation (power, off-resonance, and frequency). Phantom scans utilized 4% agar, fat, and doped water. After optimization, initial volunteer images were collected on a clinical 1.5T system (HDx, GE, Waukesha, WI) using: FOV = 64 × 32 × 32 cm3, 2.0 mm isotropic spatial resolution, TR/TE1/TE2 = 5.6/1.32/3.32 ms, α = 4°, free-breathing: scan time = 10 min, 50% acceptance window (bellows), number of projections = 39,000. In-vivo experiments utilized a 1600°, 20 ms Hamming-windowed Sinc pulse applied every 10 TRs. This pulse was applied at 210 Hz off-resonance providing some fat-saturation. In addition, two full echoes (TE1 and TE2) at ± 62.5 kHz were added to further remove fat signal while increasing SNR of water images. Twenty cardiac time frames were reconstructed using iterative soft thresholding of temporal differences with a spatial wavelet transform.

## Results

**Figure 1:**
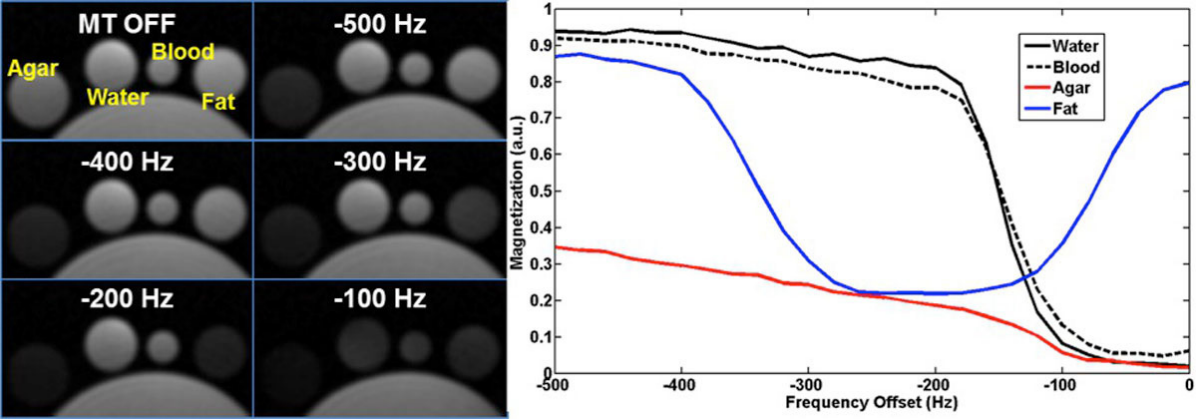
**Left: MT-prepared VIPR SPGR scans in phantoms with water, 4% agar, blood-mimicking fluid, and canola oil (fat) demonstrate signal saturations at various MT offset frequencies**. Right: Signal calculations over a range of frequencies show maximum fat suppression near its peak at 1.5T.

**Figure 2:**
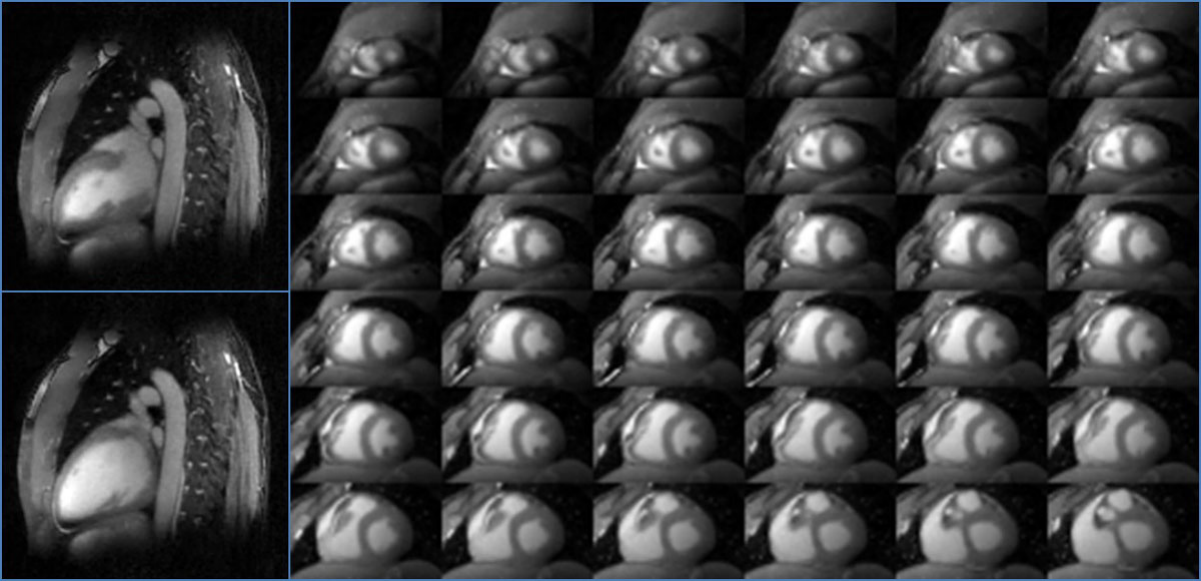
**Left: Vertical long axis reformats in end-systole (top) and end-diastole (bottom) display excellent suppression of fat and muscle without off-resonance induced banding artifacts seen in bSSFP**. Right: End-systolic short-axis stack from apex to base displays benefits of isotropic spatial resolution for retrospective reformatting of the entire heart in any orientation.

Figure 1 shows images from phantom scans for a sweep of MT off-resonance frequencies and demonstrates the potential for simultaneous suppression of muscle (agar) and fat. In-vivo results are presented in Figure 2 for two reformats: vertical long axis in end-systole and end-diastole (left) and an end-systolic base to apex short axis stack (right). Excellent blood pool to myocardium contrast and fat suppression are observed. Isotropic spatial resolution allows for retrospective whole-heart reformats in any orientation.

## Conclusions

The feasibility of a novel whole-heart functional cardiac acquisition using MT preparation with isotropic spatial resolution in a clinically reasonable scan time is presented. Further studies on optimization of acquisition parameters, including off-resonance frequency, number of projections, and acquired spatial resolution, will improve the applicability of the sequence for clinical situations.

## Funding

NIH grant 2R01HL072260.

## References

[B1] Brittain JH MRM 1998

[B2] Henkelman RM NMR Biomed 2001 10.1002/nbm.683 11320533

[B3] Barger AV MRM 2000 10.1002/1522-2594(200012)44:6<821::aid-mrm1>3.0.co;2-s 11108617

